# Characterization of Electrical Activity in Post-myocardial Infarction Scar Tissue in Rat Hearts Using Multiphoton Microscopy

**DOI:** 10.3389/fphys.2018.01454

**Published:** 2018-10-17

**Authors:** Iffath A. Ghouri, Allen Kelly, Simona Salerno, Karin Garten, Tomas Stølen, Ole-Johan Kemi1, Godfrey L. Smith

**Affiliations:** ^1^Institute of Cardiovascular & Medical Sciences, University of Glasgow, Glasgow, United Kingdom; ^2^Department of Circulation and Medical Imaging, St. Olav’s Hospital, Norwegian University of Science and Technology, Trondheim, Norway

**Keywords:** myocardial infarction, optical mapping, two-photon microscopy, intracellular calcium, border zone

## Abstract

**Background:** The origin of electrical behavior in post-myocardial infarction scar tissue is still under debate. This study aims to examine the extent and nature of the residual electrical activity within a stabilized ventricular infarct scar.

**Methods and Results:** An apical infarct was induced in the left ventricle of Wistar rats by coronary artery occlusion. Five weeks post-procedure, hearts were Langendorff-perfused, and optically mapped using di-4-ANEPPS. Widefield imaging of optical action potentials (APs) on the left ventricular epicardial surface revealed uniform areas of electrical activity in both normal zone (NZ) and infarct border zone (BZ), but only limited areas of low-amplitude signals in the infarct zone (IZ). 2-photon (2P) excitation of di-4-ANEPPS and Fura-2/AM at discrete layers in the NZ revealed APs and Ca^2+^ transients (CaTs) to 500–600 μm below the epicardial surface. 2P imaging in the BZ revealed superficial connective tissue structures lacking APs or CaTs. At depths greater than approximately 300 μm, myocardial structures were evident that supported normal APs and CaTs. In the IZ, although 2P imaging did not reveal clear myocardial structures, low-amplitude AP signals were recorded at discrete layers. No discernible Ca^2+^ signals could be detected in the IZ. AP rise times in BZ were slower than NZ (3.50 ± 0.50 ms vs. 2.23 ± 0.28 ms) and further slowed in IZ (9.13 ± 0.56 ms). Widefield measurements of activation delay between NZ and BZ showed negligible difference (3.37 ± 1.55 ms), while delay values in IZ showed large variation (11.88 ± 9.43 ms).

**Conclusion:** These AP measurements indicate that BZ consists of an electrically inert scar above relatively normal myocardium. Discrete areas/layers of IZ displayed entrained APs with altered electrophysiology, but the structure of this tissue remains to be elucidated.

## Introduction

Interruption of myocardial blood flow due to narrowing or blocking of coronary arteries leads to cell damage and death in the areas of tissue supplied by these vessels. Following this myocardial infarction (MI), ischemia of the underlying muscle causes necrosis of cardiomyocytes and vasculature. Over 2 to 3 weeks, the necrotic tissue is replaced by a fibrotic scar maintained by new vasculature. For some time, the fibrous scar tissue was thought to be inert, merely serving as a mechanical patch to prevent rupture (Lodge-Patch, 1951). A large body of evidence now exists demonstrating that the scar tissue is metabolically active and a potential target for therapeutic intervention (Cleutjens et al., 1999; Sun et al., 2002; Rog-Zielinska et al., 2016). Cross-linking collagen fibers make up the majority of the scar, forming a thinner wall than the original myocardium. Myofibroblasts populate the scar as part of the healing process, regulating collagen turnover, as well as possessing the capability to generate sustained mechanical force (Van Den Borne et al., 2010). The gross structure of the scar can vary depending on (i) the anatomical position of the occluded vessel, (ii) the extent of collateral circulation, and (iii) the transmural bias of the coronary circulation (Maxwell et al., 1987). Even after complete occlusion of a coronary artery, the subsequent loss of myocardium is not 100%. Sparse regions of residual myocardium survive through the healing process and can be detected in the scar embedded in fibrous tissue, both histologically (Camelliti et al., 2004; Walker et al., 2007) and using live tissue imaging techniques, including optical coherence tomography and two-photon microscopy (Goergen et al., 2016).

The influence of scar tissue on electrical conduction in the heart has yet to be fully elucidated. There is *in vitro* evidence of myofibroblasts electrically coupling with myocytes in co-cultured preparations (Chilton et al., 2007; Zlochiver et al., 2008; Rohr, 2009; Vasquez et al., 2010; Nguyen et al., 2012), suggesting myoblast/myocyte coupling may occur in scar tissue. Recent studies from transgenic mice have also implicated myocyte/fibroblast coupling in supporting electrical conduction through the border (BZ) and infarct zones (IZ) of the intact heart (Benamer et al., 2013; Mahoney et al., 2016; Rubart et al., 2018). While myocyte/fibroblast coupling may facilitate infarct scar conduction, the ability of such nonexcitable cells to conduct electrical impulses on their own is limited, with the spatial extent of electrical conduction known to be approximately 300 μm (Gaudesius et al., 2003), suggesting conduction through the infarct is still reliant on the presence of remnant myocardium. The degree to which the remnant myocardium is linked to the noninfarcted regions has only recently become clearer. Optical mapping studies of rabbit heart 8 weeks post-MI reported electrical signals from scar tissue that were entrained with the surviving myocardium, suggesting active remnant myocytes (Walker et al., 2007; Saba et al., 2008). In addition, mapping of hearts post-MI using electrogram (EGM) measurements revealed scar tissue characterized by low voltage, fractionated potentials in human patients (Cuculich et al., 2011) and canines (Gardner et al., 1985), indicating a degree of residual electrical activity within the scar tissue; albeit with slow, discontinuous, and nonuniform conduction, and thus a potential source of arrhythmogenic pathways (De Bakker et al., 1993; Crawford et al., 2010).

The objective of this study was to examine the electrical activity of a stable MI scar that developed after complete occlusion of a major coronary artery. This represents the most extreme situation clinically, where a minimum of myocardial tissue is expected to survive. Optical techniques were used to measure electrical activity, enabling membrane potential changes to be studied noninvasively. Our data shows distinct structure/function differences between normal zone (NZ), BZ, and IZ areas of myocardium that have not been seen hitherto in intact hearts.

## Materials and Methods

All procedures were carried out in accordance with the UK Animals (Scientific Procedures) Act 1986 and conformed to the Guide for the Care and Use of Laboratory Animals (National Institutes of Health publication No. 85-23, revised 2011). Ethical approval for all procedures was granted by the local Glasgow University Ethical committee and the UK Home Office. All chemicals were purchased from Sigma-Aldrich, UK unless otherwise stated. Fluorescent dyes were purchased from Biotium (Hayward, CA, United States) and Thermo-Fisher (Waltham, MA, United States).

### Animal Model

**FIGURE 1:**
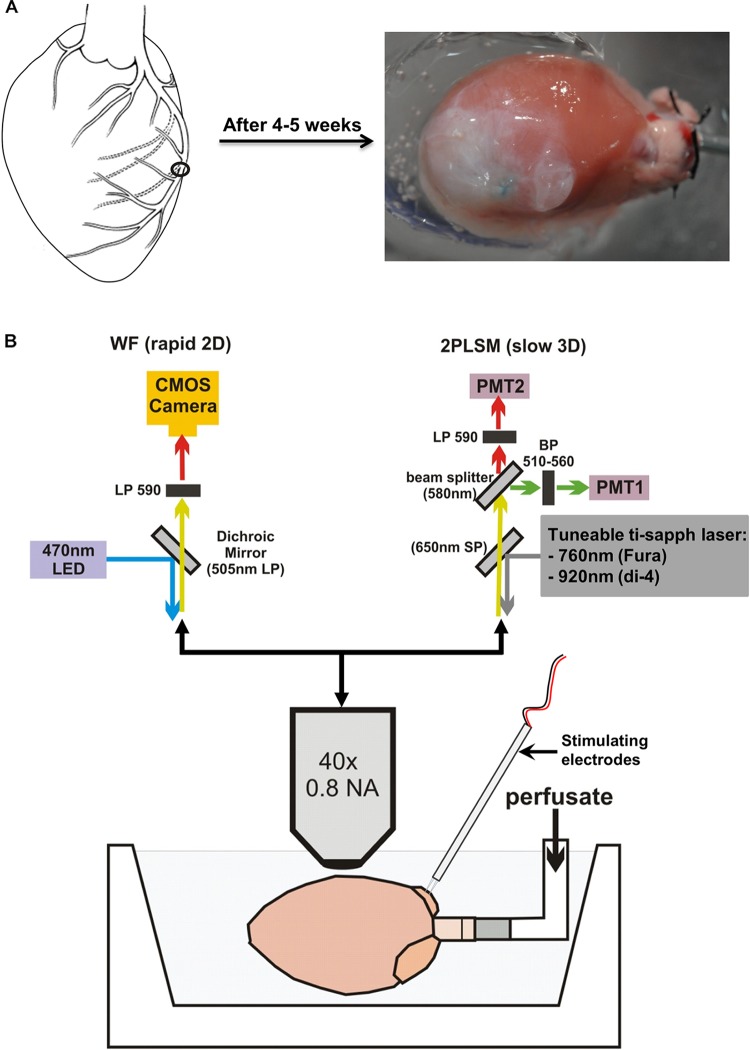
Infarct development and microscope setup. **(A)** Schematic diagram showing the permanent surgical ligation performed on the left anterior descending coronary artery. Four to five weeks post-surgery, a large infarct is produced. **(B)** Langendorff-perfused hearts were positioned horizontally and imaged using an upright microscope. Optical settings were switched between a widefield (WF) system for rapid 2D imaging (top left) and a 2-photon laser scanning microscopy (2PLSM) system for slow 3D imaging (top right).

MI was induced in male Wistar rats (weight 225–250 g, *n* = 10) by permanent ligation of the left anterior descending coronary artery. Four to five weeks post-procedure a clear IZ was produced (**Figure 1A**). An additional six animals were also used for a subset of experiments, bringing the total number of animals used in the study to 16.

### Whole-Heart Langendorff Preparation

Four to five weeks after surgery, rats were euthanized by cervical dislocation. The heart was quickly excised and placed in ice-cold modified Tyrode’s solution of composition (in mmol/L) 93 NaCl, 20 NaHCO_3_, 1 Na_2_HPO_4_, 1 MgSO_4_, 5 KCl, 1.8 CaCl_2_, 20 Na-acetate, 20 glucose. Hearts were mounted via the aorta onto a cannula and retrogradely perfused at 9 ml/min using the same Tyrode’s solution at 37°C with pH maintained at 7.4 by bubbling with a 95% O_2_/5% CO_2_ gas mixture. Perfusate was then switched to a Tyrode’s solution containing 10 mmol/L 2,3-butanedione monoxime (BDM) and 10 μmol/L blebbistatin (Enzo Life Sciences, Exeter, United Kingdom) to inhibit contraction and minimize movement artifacts. The heart was positioned horizontally in a custom-built Perspex chamber to enable imaging of the left ventricle (LV) (**Figure 1B**). A pseudo-electrocardiogram (ECG) was monitored throughout the experiment using 2 Ag/AgCl disk electrodes placed close to the heart, with a reference electrode placed in the perfusion bath. Two platinum electrodes connected to an isolated stimulator were positioned on the right atrial (RA) appendage to enable pacing of the hearts via the physiological endocardium to epicardium conduction pathway, at a cycle length of 200 ms (5 Hz).

### Optical Measurements of Voltage in NZ, BZ, and IZ

Optical measurements were made as described in detail previously (Kelly et al., 2013). Briefly, hearts were loaded with a 50 μL bolus of 2 mM voltage-sensitive dye di-4-ANEPPS over a 10 min period. Widefield single-photon epifluorescence recordings from the LV were made using a CardioCMOS-SM128 camera (Redshirt Imaging, Decatur, GA) with a 590 nm long pass emission filter. Excitation was provided by LED light at 470 nm. Image resolution was 128 × 128 pixels/frame, and recordings were made at a frame rate of 2.5 kHz. Two photon (2P) laser scanning microscopy (2PLSM) was carried out using a Zeiss LSM 510 NLO upright microscope (Carl Zeiss, Jena, Germany) equipped with a Ti:Sapphire 690–1080 nm tunable laser (Chameleon Ultra II, Coherent, Santa Clara, CA). These 2P measurements provided a high degree of depth resolution, enabling identification of the discrete tissue layers exhibiting electrical activity (Rubart, 2004). Di-4-ANEPPS was excited at 920 nm, with emission collected by two bi-alkali PMT detectors at 510–560 nm and 590–650 nm, respectively, enabling ratiometric measurements to be made. Line scans, with a scan time of 0.39 ms for short scans and 1.93 ms for long scans, were performed in the direction of cell orientation observed at the epicardial surface. Line scanning was initiated following the arrival of a trigger pulse, synchronized by the electrical stimulus pulse used to pace the hearts. A diagram of the widefield and two photon imaging setup is shown in **Figure 1B**.

Sequential widefield and 2P voltage recordings were made in myocardium remote from the infarct scar in the NZ, in the BZ at the edge of the visible scar, adjacent to the NZ, and within the IZ toward the center of the scar. Widefield electrical mapping using a 10×/0.3 NA objective lens (Carl Zeiss, Jena, Germany) was first used to identify the areas of remnant electrical activity within the BZ and IZ. Widefield electrical mapping was then repeated on regions displaying electrical signals with a 40×/0.8 NA objective (Carl Zeiss, Jena, Germany) to further localize electrically active areas. Finally, structures in the optical plane were imaged using 2P excitation in frame scan mode, then electrical signals recorded using line scan mode at discrete depths below the epicardial surface. A series of recordings were made, starting at 50 μm below the surface and at increasing depths (50–100 μm steps) until the signal-to-noise ratio (S/N) became too low to distinguish a clear action potential (AP) signal. The ability to visualize clear structures decreased at deeper layers; the maximum depth from which discernible images could be obtained depended on the zone, but electrical signals from line scan recordings could always be recorded beyond the layers where structures could be imaged. It was therefore not possible to identify the source of electrical activity directly from images of tissue structure at the deeper layers. A subset of experiments was performed using a modified upright 2P laser scanning setup (Intelligent Imaging Innovations; Denver, CO) utilizing a pair of high sensitivity GaAsP PMT detectors, and using a combination of FluoVolt and Rhod2AM, excited at 840 nm (see **Supplementary Methods** for full details). These were used to verify findings in a higher sensitivity setup.

### Determining the Source of Electrical Activity in the Scar

To determine the cellular origin of electrical activity in the scar, measurements of intracellular Ca^2+^ were made in regions where voltage signals were measured by prior loading of the myocardium with Fura-2/AM. If the signals were arising from residual myocytes Ca^2+^ transients (CaTs) would be expected in response to an electrical stimulus. However, if the voltage signals were arising from abnormal myocytes or other cellular entities (i.e., fibroblasts or myofibroblasts), CaTs may not be produced in response to electrical stimulation (Chilton et al., 2007).

Tyrode’s solution for these experiments was supplemented with 1 mmol/L probenecid to block anion transporters that excrete Fura-2, thus improving dye retention in the cell (Di Virgilio et al., 1988). Fura-2/AM was prepared as a 1 mmol/L stock in DMSO-pluronic acid F-127 (25% w/v). A 100 μL bolus of dye was injected into a bubble trap in the perfusion line to allow dilution of the dye and slow loading into the heart. Additional boluses of dye were injected if required due to low or time-dependent loss of fluorescence signal. Fura-2/AM was 2P excited at 760 nm, and fluorescence emission was directed through a short-pass 650 nm dichroic mirror and collected at 510–560 nm. Ca^2+^ measurements were made immediately after any voltage signals were detected in the same plane of focus and using the same scan line and 1.93 ms scan time.

### Data Analysis and Interpretation

Widefield voltage signals were averaged from 3 × 3 pixel arrays. 2P voltage and Ca^2+^ signals were processed using custom written software that utilized the information from exact cycle length times and line scan rates to align and produce an averaged voltage or Ca^2+^ trace from 25 sequential stimuli (5 s of recordings). AP signal characteristics were analyzed from the averaged trace. These measurements included 10–90% upstroke rise time (TRise) and duration of the AP from 50% activation to 50, 75, and 90% repolarization (APD50, APD75, and APD90, respectively). Activation times were determined as the time of arrival of the AP at that point in the LV wall relative to the time of stimulation.

S/N was calculated as the peak amplitude of the whole trace following a single stimulus pulse (signal) divided by the peak amplitude of the trace during the diastolic period (noise) (**Supplementary Figure S1**). An S/N value of 1 indicated no signal over and above the noise of the baseline. Based on observations of individual traces and corresponding S/N values, an S/N > 1.4, was considered a meaningful transient signal (voltage or Ca^2+^). All traces with S/N > 1.4 were further scrutinized to rule out artefactual signals produced by movement or noise spikes. All data are expressed as mean ± standard error. Groups of data were compared using Student’s *t*-test.

## Results

### Initial Identification of Areas of Electrical Activity Using Widefield Optics

**FIGURE 2:**
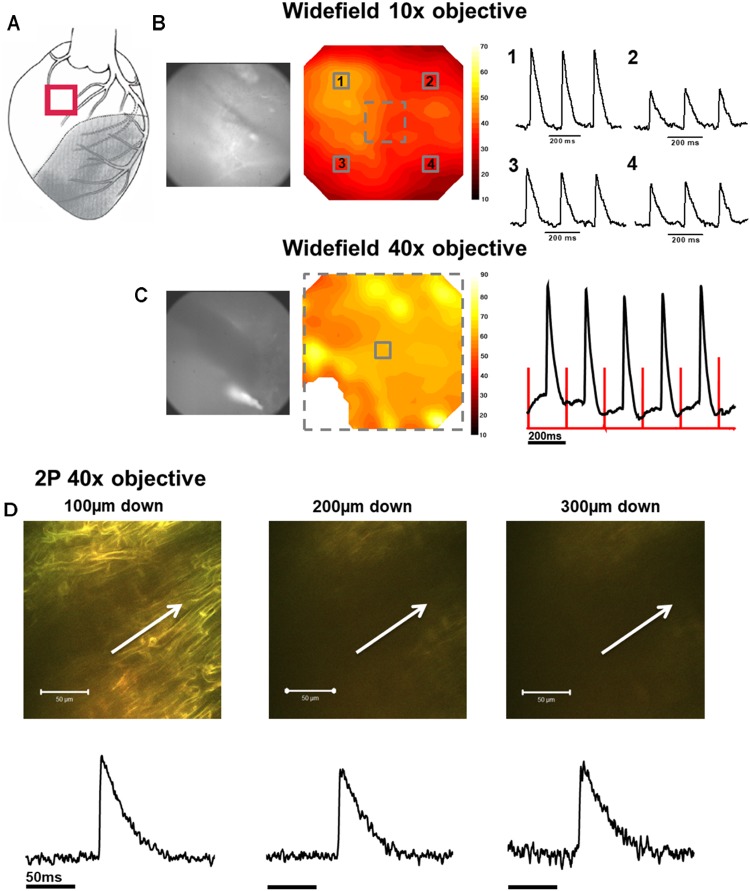
Widefield and 2P signals in the normal zone (NZ). **(A)** Schematic diagram of the infarcted heart. The red square indicates the approximate location of the measurements in the NZ. **(B)** Widefield measurements were first taken from NZ using a 10× objective. CMOS camera image (left) and signal-to-noise ratio contour map (middle) of di-4-ANEPPS fluorescence in the NZ. Line traces on the right show action potentials (APs) from 3 × 3 pixel averaged regions (indicated by numbered solid squares on contour map). **(C)** Measurements were focused to a smaller area using a 40× objective [indicated by the dashed square on contour map in **(B)**]. A CMOS camera (left) image and signal-to-noise ratio contour map (middle) taken from the NZ at 40× magnification. An area in the bottom left of the contour map field was removed due to movement artifact distorting the signal. Right panel: 3 × 3 pixel average of the AP signal in the center of the optical field (defined by the solid square in the contour map). The red lines indicate the timing of the stimulus pulse. **(D)** A series of 2P measurements were made through increasing depth in the same optical field with the 40× objective. Shown here are 2P images and corresponding averaged APs taken (from left to right) 100, 200, and 300 μm below the tissue surface. White arrows in images indicate approximate length and direction of line scans. All the APs shown are an average of 25 APs recorded over a period of ∼5 s.

**FIGURE 3:**
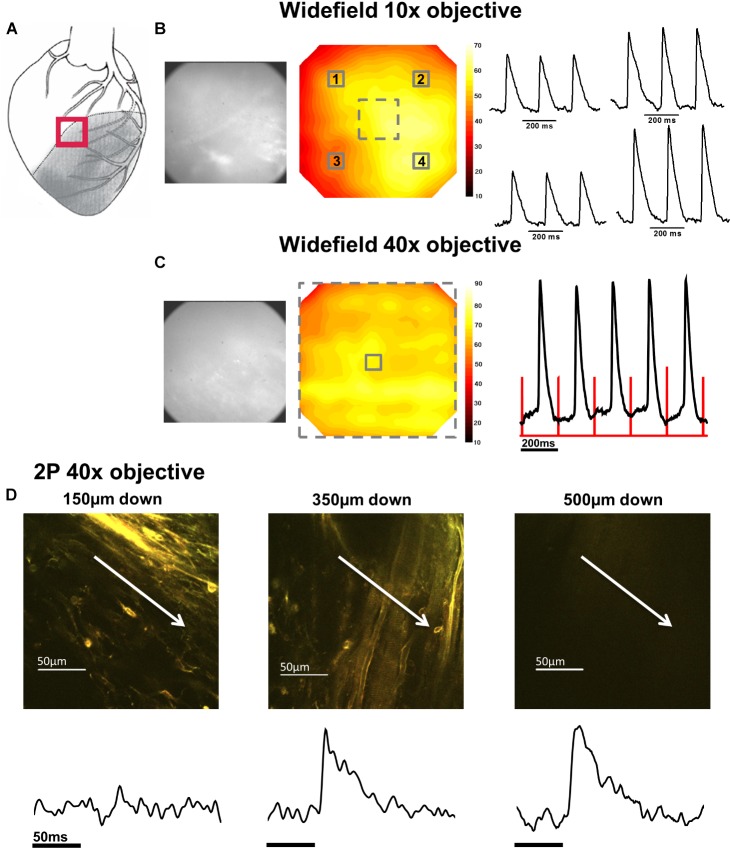
Widefield and 2P signals in the border zone (BZ). **(A)** Schematic diagram of the infarcted heart. The red square indicates the approximate location of the measurements in the BZ. **(B)** Widefield measurements taken from BZ tissue using a 10× objective. From left to right; CMOS camera image of di-4-ANEPPS fluorescence; contour map of di-4-ANEPPS signal-to-noise; 3 × 3 pixel averages (indicated by numbered solid squares in contour map) of action potentials (APs). **(C)** Measurements were then focused to a smaller area using a 40× objective [indicated by the dashed square on contour map in **(B)**]. From left to right; CMOS camera image of di-4-ANEPPS fluorescence in the BZ; Contour map of signal-to-noise in the 40× field of view; 3 × 3 pixel average of the AP signal in the center of the optical field, defined by the solid square in the contour map – red lines indicate timing of the stimulus pulse. A series of 2P measurements were made through increasing depth in the same optical field with the 40× objective. **(D)** 2P images and corresponding voltage measurement taken at (from left to right) 150, 350, and 500 μm below the epicardial surface. Myocardial structures could no longer be identified in 2P images at 500 μm; however, electrical activity was still observed at this depth. White arrows in images indicate approximate length and direction of line scans. All the APs shown are an average of 25 APs recorded over a period of 5 s.

**FIGURE 4:**
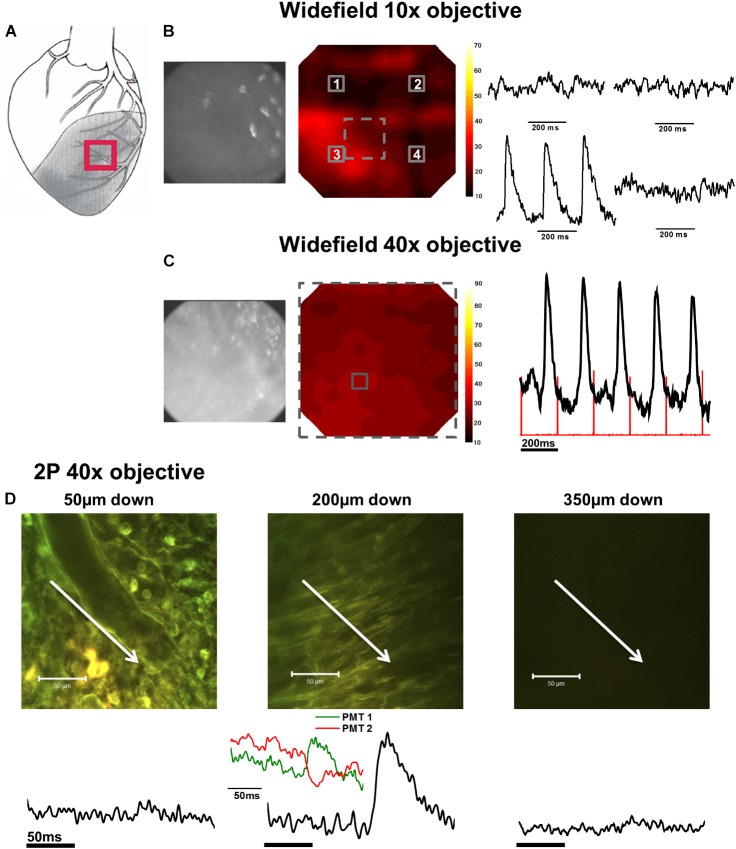
Widefield and 2P signals in the infarct zone (IZ). **(A)** Schematic diagram of the infarcted heart. Red square indicates the approximate location of the measurements in the IZ. **(B)** Widefield measurements taken from the IZ using a 10× objective. From left to right; CMOS camera image of di-4-ANEPPS fluorescence; contour map of di-4-ANEPPS signal-to-noise; 3 × 3 pixel averages (indicated by numbered solid squares in contour map) demonstrated absence of signal in all but one region of the infarct. **(C)** Measurements were then focused to a smaller area using a 40× objective [indicated by the dashed square on the contour map in **(B)**]. From left to right; CMOS camera image of di-4-ANEPPS fluorescence in the IZ; contour map of signal-to-noise in the 40× field of view; 3 × 3 pixel average of the AP signal defined by the solid square in the contour map – red lines indicate timing of the stimulus pulse. **(D)** 2P images and the corresponding voltage signal taken at (from left to right) 50, 200, and 350 μm below the tissue surface. At 200 μm below the tissue surface, tissue structure appears more organized and AP waveforms with slow activation times and upstrokes become apparent from the fluorescence ratio. Closer inspection of the individual PMT signals (inset) demonstrate an increase in fluorescence at the shorter waveband and a decrease in the longer waveband, eliminating the possibility of the fluorescence ratio signal being due to movement artefact. White arrows in images indicate approximate length and direction of line scans. All the APs shown are an average of 25 APs recorded over a period of 5 s.

In the first phase of the protocol, widefield di-4-ANEPPS fluorescence measurements were made within the NZ (**Figure 2A**), BZ (**Figure 3A**), and IZ (**Figure 4A**) with a 10× objective to identify regions of electrical activity over an area of 800 × 800 μm (**Figures 2B, 3B, 4B**). This approach was particularly important for measurements within the IZ, as not every recording indicated an electrically active region. Contour maps of S/N were constructed to allow comparison of the relative uniformity of the electrical signal in the NZ, BZ, and IZ (**Figures 2B, 3B, 4B**, respectively). However, in contrast to NZ and BZ, the IZ displayed only limited regions with AP signals of significant amplitude; large areas of IZ surface had no observable electrical activity (**Figure 4B**).

In the second phase of the protocol, the S/N across a sub-area (250 × 250 μm) of the original field was further examined using a 40× objective (**Figures 2C, 3C, 4C**) to pinpoint electrically active regions. In the case of the NZ and the BZ, the relative uniformity of the AP signal recorded with the 10× objective allowed 40× imaging to be made routinely in the center of the field of view. However, due to the nonuniformity of signal in the IZ, the area with the AP signal with the greatest S/N using the 10× objective was chosen for further examination with the 40× objective.

### Detailed Examination of Electrical Activity With 2PLSM

In the third phase of the protocol, the variation of the AP signal with depth was examined in a limited central region of the 40× field using 2PLSM. Line scan recordings of APs based on the ratio of di-4-ANEPPS fluorescence were made starting at 50 μm from the epicardial surface and every 50 μm until 500 μm from the surface. In some preparations, the CaT was also recorded from the Fura-2 fluorescence.

### AP and CaT Characteristics in NZ

**FIGURE 5:**
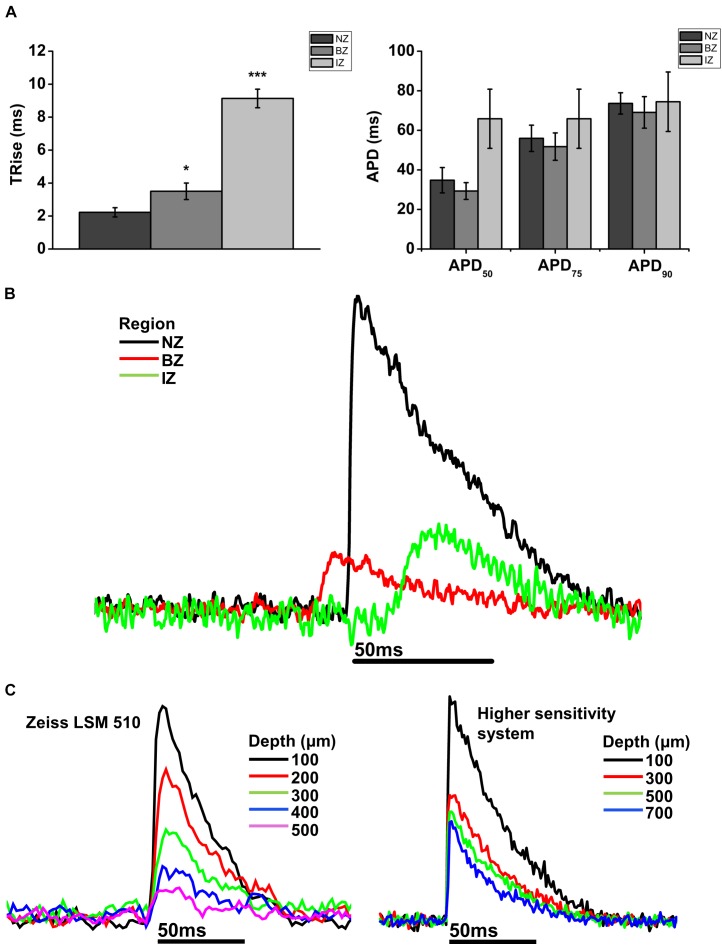
Comparison of AP parameters in normal zone (NZ), border zone (BZ), and infarct zone (IZ). **(A)** Mean 10–90% upstroke rise time (TRise) and mean action potential duration (APD) at 50, 75, and 90% repolarization obtained from 2P signals for each heart in each region. NZ, *n* = 6; BZ, *n* = 4, IZ, *n* = 3. ^∗^*p* < 0.05; ^∗∗∗^*p* < 0.001. **(B)** Representative traces from a rat MI heart showing action potentials recorded using 2PLSM from three imaging zones: Normal zone (NZ), borderzone (BZ), and infarct zone (IZ). AP upstroke was slowed in both BZ and IZ compared to NZ, with no obvious difference in APD. **(C)** Transmural action potentials recorded using two microscope systems: Zeiss LSM 510 (left) and a microscope system featuring optimized detection and optical configuration (right). Note there was no change in AP morphology with either imaging system over the depth range investigated.

**FIGURE 6:**
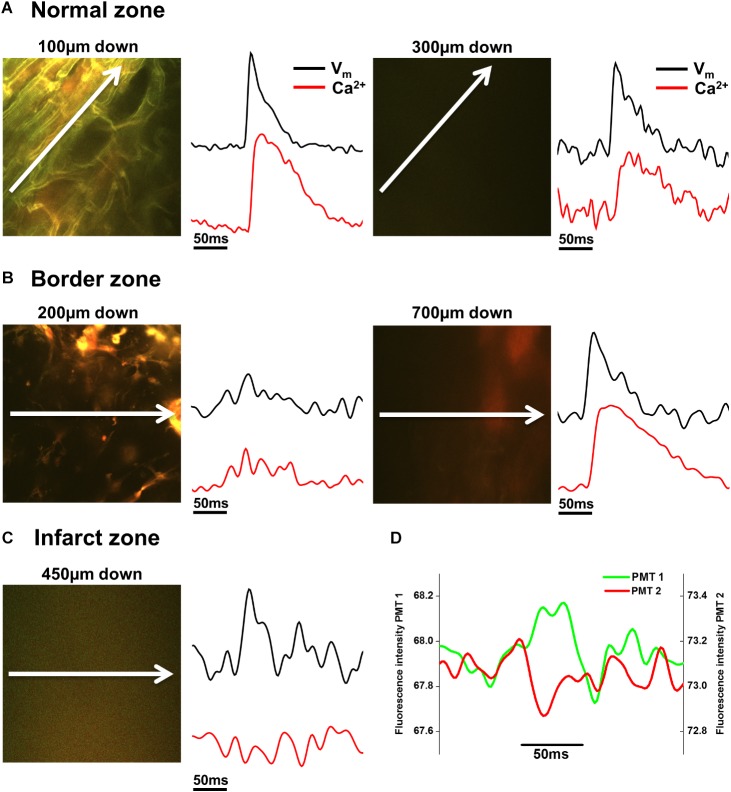
Voltage and Ca^2+^ measurements in normal zone (NZ), border zone (BZ), and infarct zone (IZ). **(A)** 2P image with corresponding voltage (Vm) and intracellular calcium (Ca^2+^) transients taken from the NZ 100 μm (left panels) and 300 μm (right panels) below the tissue surface. Vm and Ca^2+^ signals were of normal morphology in these areas. **(B)** In the BZ, toward the surface (here at 200 μm down), structures appear to resemble connective tissue and no Vm or Ca^2+^ signals were apparent (left panels). However, deeper into the tissue (700 μm down in this example) fluorescence signal with no defined structures can be detected in the 2P image, and Vm and Ca^2+^ signals with good signal-to-noise were recorded (right panels). **(C)** Within the IZ, in this example, no structures could be resolved in the 2P image at 450 μm down. However, low amplitude voltage signals were detected, with no detectable Ca^2+^ signal in the same region (left panels). **(D)** The individual PMT signals indicate that the voltage signals are not arising from movement artefact, as short and long wavelength signals are not the same. White arrows indicate length and direction of line scan. Vm and Ca^2+^ signals shown are the average of 25 APs/Ca^2+^ transients recorded over a period of 5 s.

In NZ remote from the infarct, APs had a normal time course at all depths (**Figure 2D**). Myocardial structure and electrical activity could be imaged from 50 μm below the epicardial surface, and AP morphology was unchanged through depth. However, the signals became progressively smaller due to the reduction of fluorescence signal with increasing depth (**Figure 5C**). The limit for recordable images was ∼300 μm, and the limit for AP recordings was ∼500 μm; this was increased to ∼700 μm with the higher sensitivity system. CaT signals, when recorded alongside AP signals, were detected at equivalent depths and were of consistent shape (**Figure 6A**). The amplitude of CaT signal decreased in parallel with the AP signal with increasing distance from the epicardial surface.

### AP and CaT Characteristics in BZ

Measurements from BZ area, that is, the scar adjacent to the NZ, revealed no AP signals from the layers immediately below the surface (50–100 μm depth), but AP signals were recorded at deeper layers (**Figure 3D**). The exact depth at which electrical signals were first identified varied between preparations but was within the range of 150–300 μm. Normally, AP signals could be detected deeper into the tissue than in the NZ; in two hearts this was up to 1000 μm below the epicardial surface. This indicated that in the scar bordering the NZ, there are layers of connective tissue overlying normal myocardium. The averaged APs recorded in the BZ had a similar time course for repolarization parameters but slower rise times than NZ signals (**Figure 5A**) (3.50 ± 0.50 ms vs. 2.23 ± 0.28 ms, *p* < 0.05). This can also be observed in superimposed traces from NZ, BZ, and IZ from an example heart (**Figure 5B**). Activation times were measured from both widefield and 2P AP signals. Widefield measurements of activation delay between NZ and BZ showed no difference (3.36 ± 1.55 ms, *p* = 0.41) indicating absence of a significant activation delay in the BZ. In hearts where CaTs were recorded, the appearance of these signals paralleled AP signals in terms of the depth at which they were detected and the maximum depth that they could be recorded (**Figure 6B**).

### AP and CaT Characteristics in IZ

AP and CaT characteristics in IZ were only examined where electrical signals were detected using widefield imaging of di-4-ANEPPS with the 40× objective. This was possible in 5 out of 10 hearts in this configuration. The electrical activity in the IZ differed from that in BZ, as it was only detected in thin layers of tissue approximately 100–300 μm deep and was not found deeper within the tissue (**Figure 4D**). AP morphology was different from that found in NZ (**Figure 5B**), with longer rise times than NZ (9.13 ± 0.56 ms vs. 2.23 ± 0.28 ms, *p* < 0.001; **Figure 5A**). Closer inspection of the fluorescence signals from each channel revealed that this difference in morphology was not due to movement artifact, as the signals collected between 510 and 560 nm (Channel 1) increased upon depolarization but decreased at Channel 2 (collected between 590 and 650 nm), indicating a genuine voltage signal (**Figure 4D** – inset graph in middle panel and **Figure 6D**). Activation times of the widefield signals showed heart-to-heart variation. In two hearts, there were no delays in activation times in IZ relative to NZ. In one heart, there was moderate delay (7 ms) and, in one heart, there was substantial delay (40 ms); mean activation delay was 11.88 ± 9.43 ms. It was not possible to compare activation times in one heart due to differences in the stimulation mode of recordings from NZ and IZ. This variation in activation delay was also observed in the AP signals recorded through depth with 2P excitation. Measurements of AP duration at 50% repolarization (APD_50_) showed a trend (not significant) toward longer duration in IZ compared to NZ (**Figure 5A**). In contrast to the NZ and BZ, discrete layers within the IZ at which APs were recorded did not show detectable CaTs (**Figure 6C**).

### Signal-to-Noise Profile in the NZ, BZ, and IZ

**FIGURE 7:**
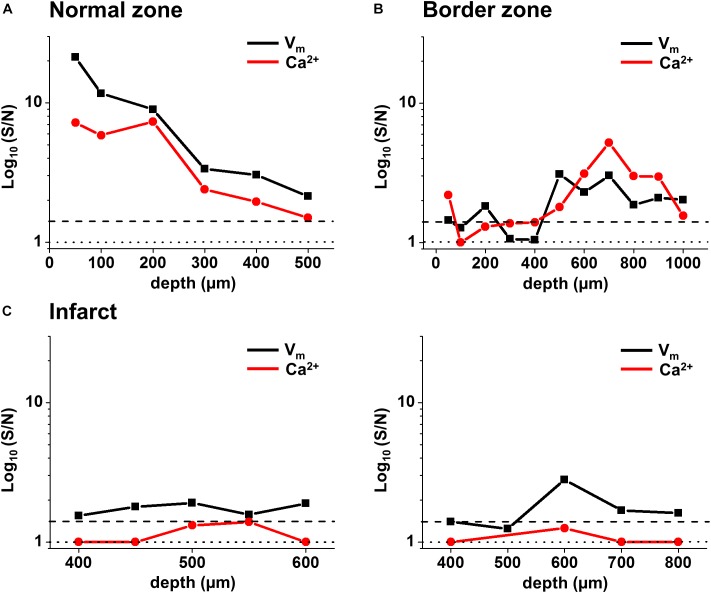
Voltage and Ca^2+^ signal-to-noise in the normal zone (NZ), border zone (BZ), and infarct zone (IZ) from individual hearts. Voltage (black) and Ca^2+^ (red) S/N were plotted through depth for individual hearts in **(A)** NZ, **(B)** BZ, and **(C)** IZ. S/N in the IZ for two hearts [left and right panels in **(C)**] were plotted between depths where a voltage signal was detected. S/N values of 1 (no distinguishable signal) are illustrated by the dotted line. S/N values of 1.4 (indicating a real signal) are illustrated by the dashed line.

Values of S/N for voltage and Ca^2+^ signals were plotted against depth for each zone (NZ, BZ, and IZ – **Figure 7**). Plots from a single heart are shown to highlight the characteristics of the signals in the three zones. A distinctive profile of S/N for remote (NZ) tissue was apparent, in which the highest S/N was obtained close to surface, with decreasing S/N through depth. Measurements were not made deeper than 500–600 μm below the surface as the S/N values were close to the detection threshold (1.4). This S/N profile was similar for both voltage and Ca^2+^ signals.

In the BZ, the S/N values of fluorescence signals were <1.4 at the surface indicating the absence of detectable AP signals, but the values of S/N increased deeper into the tissue (to levels similar to those measured in NZ), and remained higher than in remote tissue at 500–1000 μm depth. Again, a similar profile was obtained for both voltage and Ca^2+^signals. At the deepest layers where no signal could be detected, S/N of recordings was comparable to those obtained in the absence of excitation light (**Supplementary Figures S2A,B**). This indicates that the absence of signal was due to the lack of penetration of excitation light deep into the tissue.

**FIGURE 8:**
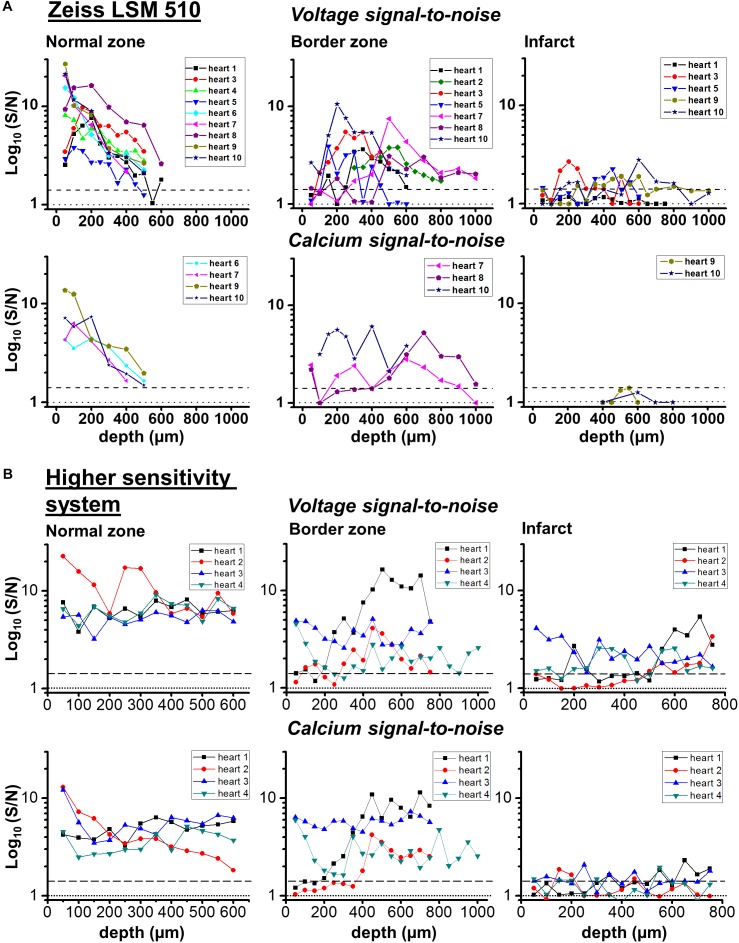
Transmural signal-to-noise in remote, border zone and infarct regions – voltage vs. calcium. S/N values (log10) for voltage (upper row) and calcium (lower row) as a function of tissue depth for individual hearts in two microscope systems: **(A)** Zeiss LSM 510 and **(B)** microscope system featuring optimized detection and optical configuration. From right to left; remote myocardium; border zone; infarct. For border and infarct zones, traces are displayed from hearts which demonstrated significant S/N (>1.4) constituting detection of a signal. Dashed line for each graph indicates threshold for signal detection.

In the IZ, despite a significant widefield signal, the S/N values in the majority of layers were below the value of 1.4 indicating no detectable excitable tissue. However, there were discrete layers within the scar in which voltage S/N exceeded 1.4, but a significant CaT could not be recorded at any of these locations. This profile of S/N was consistent in each of the three zones in all the hearts examined for this study (**Figure 8A**). Very similar results were obtained using a higher sensitivity 2P laser scanning system with improved optics and detector sensitivity (**Figure 8B**). In a subgroup of experiments, improved signal collection allowed significant AP signals to be detected in various subepicardial layers from infarcted tissue in four out of six hearts (**Figure 8B** – top right panel). In a few cases, this improved signal detection also recorded Ca^2+^ transients (s/n ratio > 1.4) in the same layer as an AP. However, for the majority of cases, where an AP was detected within the infarct, no significant Ca^2+^ transient could be detected (**Figure 8B** – bottom right panel). Despite the improved transmural AP signal detection afforded using higher sensitivity detectors and an optimized optical configuration, improvement in deep tissue structural imaging was marginal (∼50 μm – data not shown).

## Discussion

In this study, optical APs from di-4-ANEPPS fluorescence were recorded from myocardial layers within the homogenous surviving myocardium (NZ), in comparable layers of the scar at the border to the NZ (BZ) and from within the epicardial scar area (IZ). In a sub-group of experiments, the voltage signals were recorded along with intracellular Ca^2+^ signals after loading the heart with Fura-2/AM. The aim of this approach was to identify the cellular origin of the AP using a functional correlate, where conventional imaging would have been unable to resolve structure sufficiently; a voltage signal accompanied by a CaT clearly indicated a myocardial origin of the AP. We hypothesized that a voltage signal with no accompanying CaT suggested that the source of the electrical signal could be substantially modified cardiomyocytes or fibroblasts/myofibroblasts coupled to electrically active cardiomyocytes. From the results presented, we found discrete regions of surviving myocardium within the IZ exhibiting very small voltage signals, but the lack of calcium transient was more likely due to the low S:N ratio of signals arising from such small tissue volumes deep within a scattering structure such as the infarct. Using a higher sensitivity system supported the original findings, with signals only arising from discrete infarct areas and no direct evidence of fibroblast/myofibroblast associated voltage signals.

### Characteristics of Signals in Normal Myocardium

**FIGURE 9:**
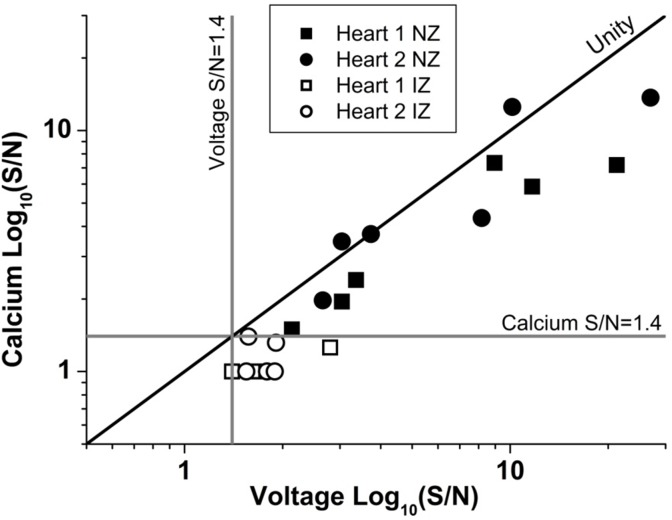
Unity of action potential and calcium transient signal-to-noise. The signal to noise of AP and CaT plotted against one another for two example hearts from normal zone (NZ) (closed symbols) and ischemic zone (IZ – open symbols). CaT signal was on average 76% of the equivalent AP signal at all depths and remained below the detection threshold for calcium signals in the IZ.

As expected, the AP signal in the NZ was homogeneous over the area imaged in widefield and through the depth of the myocardium sampled by the 2P excitation. The S/N of the optical AP decreased with increasing depth due to loss of excitation light power as a consequence of photon scattering in the intervening layers (Kelly et al., 2013; Ghouri et al., 2015), but there were no major changes in AP shape through depth and the activation sequence reflected rapid transmural propagation. In the NZ, voltage signals were detected down to a maximum depth of 500–600 μm below the epicardial surface. In each layer, Ca^2+^ transients accompanied the optical AP even at distances from the epicardial surface where there was insufficient signal to distinguish any structural detail. As shown in **Figure 9**, the S/N of both AP and CaT signals decreased from a value of approximately 30 at the epicardial surface to below the detection threshold (<1.4) at 600 μm. The unity line emphasizes that the S/N of the CaT signal was on average 76% of the equivalent AP signal at all depths (**Figure 9**). These values are a function of the dye loading protocols used in the study.

### Characteristics of Signals in the BZ

In the BZ, a layer of electrically inert tissue was present on the epicardial surface of all hearts, although the thickness of this layer varied from heart to heart. The presence of such rim structures have been reported previously from high resolution histology imaging (Walker et al., 2007). The structure observed from optical sectioning and the lack of electrical activity indicated that this was a layer of connective tissue, which in some hearts was between 300 and 400 μm thick. However, below the connective tissue layer, APs were detected with normal activation times and repolarization characteristics, but with an average time of depolarization that was 150% of the values recorded in the NZ. These electrical signals were recorded at considerably larger distances into the tissue surface than was possible in the NZ (∼1000 μm vs. ∼500 μm). The ability to image and measure optical APs at greater depths is likely due to the lower scatter of excitation light by the upper layers of connective tissue when compared to equivalent layers of compact myocardium (Mourant et al., 1998). Optical properties of normal and infarcted myocardium have been compared previously using tissue from human patients, indicating that infarct scar exhibits a significantly lower scattering coefficient compared to normal healthy myocardium (Splinter et al., 1991).

The voltage signals in the BZ were accompanied by Ca^2+^ transients at all depths. These results are consistent with histological studies of the infarct structure demonstrating the presence of a wedge of myocardium projecting approximately 1 mm into the scar beneath superficial layers of connective tissue in BZ regions (Walker et al., 2007). This increases the apparent size of the scar at the epicardial surface, but covering a volume of tissue in the mid-myocardium with relatively normal histological appearance. Increased thickness of the epicardium has been described in response to MI (Smart et al., 2011; Zhou et al., 2011; Tian et al., 2015), along with the formation of epicardium-derived cells that have both fibroblast and smooth muscle markers. These cells release angiogenic factors to stimulate the recovery of the tissue from infarction. The myocardium below the scar in the BZ had APs with a lower rate of depolarization, suggesting altered excitability of this area of myocardium either through electrical remodeling (i.e., altered sodium current and/or gap-junction function) or altered activation sequence. Walker et al. (2007) reported slowed epicardial conduction at the BZ in response to epicardial stimulation which would be consistent with the slowed AP rise-time observed in these studies. As shown previously, the rate of rise of the AP depends critically on the direction of propagation (Spach et al., 1992; Kelly et al., 2013); APs propagating longitudinally through epicardial tissue along the fiber axis have a slower rate of rise of AP due to reduced resistance of the downstream electrical network when compared to transverse propagation.

### Characteristics of Signals in the Infarct

In the IZ, only discrete areas of AP signals were identified with widefield imaging of the epicardial surface of the scar. The AP signals in the IZ were entrained to the stimulus, but the range of activation delays were higher than NZ and BZ, suggesting low conduction velocity and/or altered electrophysiology of the remnant myocardium within the scar. This supports previous findings of low conduction velocity and reduced AP amplitude that has previously been described in optical mapping studies of rabbit hearts post-MI (Saba et al., 2008). The APs recorded were of similar duration to NZ and BZ but with significantly longer rise times (∼400% compared to NZ) indicating altered excitability of this myocardium either through electrical remodeling (i.e., altered sodium current and/gap-junction function) or altered activation sequence. Unlike in the BZ, the electrically active regions of the IZ only occurred in a limited layer (100–300 μm), which suggests that the strands of surviving myocardium remain coupled to the surviving myocardium, despite their narrow dimensions.

The S/N of APs recorded from the IZ was considerably lower than at equivalent depths of NZ or BZ. This supports the idea that the recordings originate from small, possibly sparse layers of myocytes; 2P images from layers of the IZ containing AP signals showed no structural evidence of myocardium (e.g., striated tissue) and were indistinguishable from IZ layers without electrical signals. The average S/N of AP signals in the IZ was 1.75, based on the parallel recording of CaT within the rest of the myocardium (**Figure 8**); these signals would be expected to be accompanied by CaT with an S/N value of approximately 1.3, that is, lower than the threshold for signal detection. This relationship is demonstrated in **Figure 9** and indicates that the values of the AP and CaT signals from the IZ had the same relative values to that in NZ, therefore the absence of detectable CaT signals in the IZ was most likely to be a function of the low amplitude di-4-ANEPPS and Fura-2 signals originating from small volumes of myocardium. Using a higher sensitivity system with improved optics and dyes enabled electrophysiological signal detection from deeper layers in both the remote myocardium (up to 700 μm) and the infarct tissue (up to 1200 μm), revealing a population of cells within the infarct which exhibited resolvable Ca^2+^ transients (**Figure 8B**). However, the majority of AP signals arising from the infarct were not accompanied by a Ca^2+^ transient signal.

### Implications for Whole Heart Function After Myocardial Infarction

This study establishes the electrical features of myocardium on the border to the infarct and the remnant myocardium within the IZ. The combined approach of widefield and 2PLSM techniques provides new insight into the presence, abnormality, and transmural localization of functional remnant tissue within and around the scar, increasing our understanding of the potential functional consequences of this activity. Recent studies have highlighted the potential role of fibroblasts/myofibroblasts coupled to surviving myocardium as supporting electrical conduction across the infarct scar (Mahoney et al., 2016). Using a transgenic mouse model with fibroblast-specific Cx43 knockout, Mahoney et al. (2016) reported loss of electrical conduction through ventricular infarct scar tissue in the absence of Cx43 expression in fibroblasts, suggesting that fibroblast coupling is essential for remnant electrical conduction through infarcted myocardium. It is known, however, that fibroblasts can only sustain electrical conduction across a finite distance (approximately 300 μm) (Gaudesius et al., 2003), suggesting that coupled fibroblasts alone would not support electrical propagation in an infarct much larger than this. The combined presence of remnant myocardium coupled to fibroblasts could therefore be critical in bridging the gap between infarct scar and the normal, noninfarcted tissue, particularly in larger infarcts.

The nonstandard electrical activity adjacent to the surviving myocardium may increase the propensity for arrhythmic behavior by providing regions of electrical heterogeneity in terms of AP characteristics and electrical load. Conversely, the presence of entrained electrical activity within the scar may also be beneficial since conduction within the IZ may prevent a pattern of electrical activation that conducts around an electrically inert region and thus creating the potential for re-entry circuits. Furthermore, this study suggests that even in the situation where the coronary artery is occluded, and the downstream tissue is exposed to complete ischemia, 5 weeks post-MI there are still areas of the IZ with significant remnant myocardium electrically coupled to the surviving myocardium. Identifying these regions containing electrically active remnant myocardium will be important to the success of efforts to repopulate an infarct scar with myocardium using stem-cell derived cardiomyocytes (Laflamme et al., 2007; Templin et al., 2012). Directing the implanted cells toward target regions of the infarct where entrained electrical activity persists could increase the prospect of these cells synchronizing electrically with the surviving myocardium. Indeed, 2PLSM measurements of voltage and Ca^2+^ could prove a useful tool for assessing if implanted cells have formed a functional syncytium with the pre-existing myocardium (Lu and Rubart, 2014; Weinberger et al., 2016).

A limitation of the study was the inability of the optical system to provide sufficient information to allow unequivocal identification of the tissue responsible for the electrical activity arising in the IZ. The 2P excitation mode was able to image the region of myocardium directly below the thick connective tissue layer in the BZ at approximately 500μm from the epicardial surface. However, there were no obvious myocardial structures in regions of the IZ that displayed electrical activity compared to regions that did not. This may be explained by the very small amount of myocardial tissue that generates this signal and/or the distortion of the geometry of the tissue within the infarct. Despite also using a system with improved optics and enhanced signal detection capabilities, resolution of structures within the infarct and normal myocardium was only marginally improved (an increase in structural imaging depth by ∼50 μm). For future studies, mapping of the infarct and BZ regions using a modified functional approach as outlined in this study, combined with subsequent tissue clarification and structural imaging of the same regions in the optically cleared tissue using cell labels may be required to positively identify the cellular structures within the IZ responsible for the remnant electrical activity (Chudakov et al., 2010).

## Conclusion

The use of 2PLSM has demonstrated myocardial sub-surface functional electrophysiological differences between infarcted, near-infarcted, and noninfarcted regions of the intact MI heart that could not be ascertained by other means. This study has demonstrated that BZ of the infarct consists of an electrically inert scar above relatively normal myocardium and that IZ includes discrete layers displaying entrained APs, but with altered electrophysiology.

## Author Contributions

IG completed the majority of the experimental work and analysis and contributed to drafting and proofreading the manuscript. AK completed some of the experimental work and analysis and contributed to drafting and proofreading the manuscript. SS and KG completed the supplementary experimental work and analysis. TS complete the supplementary experimental work and analysis and drafting of manuscript. O-JK assisted in planning the study, in experimental work, and editing the text of the manuscript. GS planned the study, assisted in experimental work and some analysis, drafted the manuscript and diagrams.

## Conflict of Interest Statement

The authors declare that the research was conducted in the absence of any commercial or financial relationships that could be construed as a potential conflict of interest.
