# Corrigendum: Characterization of Electrical Activity in Post-myocardial Infarction Scar Tissue in Rat Hearts Using Multiphoton Microscopy

**DOI:** 10.3389/fphys.2019.00364

**Published:** 2019-04-05

**Authors:** Iffath A. Ghouri, Allen Kelly, Simona Salerno, Karin Garten, Tomas Stølen, Ole-Johan Kemi, Godfrey L. Smith

**Affiliations:** ^1^Institute of Cardiovascular & Medical Sciences, University of Glasgow, Glasgow, United Kingdom; ^2^Department of Circulation and Medical Imaging, St. Olav's Hospital, Norwegian University of Science and Technology, Trondheim, Norway

**Keywords:** myocardial infarction, optical mapping, two-photon microscopy, intracellular calcium, border zone

“Ole-Johan Kemi” was not included as an author in the published article. The corrected Author Contributions Statement and Acknowledgments Statement appears below.

## Author Contributions

IG completed the majority of the experimental work and analysis and contributed to drafting and proofreading the manuscript. AK completed some of the experimental work and analysis and contributed to drafting and proofreading the manuscript. SS and KG completed the supplementary experimental work and analysis. TS complete the supplementary experimental work and analysis and drafting of manuscript. O-JK assisted in planning the study, in experimental work, and editing the text of the manuscript. GS planned the study, assisted in experimental work and some analysis, drafted the manuscript and diagrams.

