# Supplementary material for: Characterization of Electrical Activity in Post-myocardial Infarction Scar Tissue in Rat Hearts Using Multiphoton Microscopy
Source: Front Physiol. 2018 Oct 17;9:1454. doi: 10.3389/fphys.2018.01454 (PMC6199960; doi:10.3389/fphys.2018.01454)

Supplementary materials and methods

Optical measurements of voltage and calcium using two-photon laser scanning microscopy

Isolated perfused hearts were loaded with a 50µL bolus of 2mM voltage-sensitive dye di-4-ANEPPS over a 10min period. Widefield single-photon epifluorescence recordings from the LV were made using a CardioCMOS-SM128 camera (Redshirt Imaging, Decatur, GA) with a 590nm long pass emission filter. Excitation was provided by LED light at 470nm. Image resolution was 128x128 pixels/frame and recordings were made at a frame rate of 2.5kHz. Two photon (2P) laser scanning microscopy (2PLSM) was carried out using a Zeiss LSM 510 NLO upright microscope (Carl Zeiss, Jena, Germany) equipped with a Ti:Sapphire 690-1080nm tunable laser (Chameleon Ultra II, Coherent, Santa Clara, CA). Hearts were also loaded with the intracellular Ca^{2+}-sensitive fluorophore Fura-2AM (50µL bolus of 1mM stock solution dissolved in 1mL DMSO containing 20% pluronic F-127). Di-4-ANEPPS was excited at 920nm, with emission collected by two bi-alkali PMT detectors at 510-560nm and 590-650nm respectively, enabling ratiometric measurements to be made. Fura2-AM was excited at 760nm and collected between 510-560nm. A series of sequential line scan recordings was performed, starting 50µm below the epicardial surface and moving focus through the tissue in 50µm steps, until the signal-to-noise ratio (S/N) became too low to distinguish a clear action potential (AP) signal. The ability to visualise clear structures decreased at deeper layers; the maximum depth from which discernible images could be obtained depended on the zone, but electrical signals from line scan recordings could always be recorded beyond the layers where structures could be imaged. It was therefore not possible to identify the source of electrical activity directly from images of tissue structure at the deeper layers.

A subset of experiments (n=6) were then performed using a modified upright 2P laser scanning setup (Intelligent Imaging Innovations; Denver, CO). The microscope used the same tunable Ti:Sapphire

laser, but imaging was performed using a 20x 1.0NA water immersion objective lens (Carl Zeiss, Jena, Germany) and detection was achieved using a pair of high sensitivity GaAsP PMT detectors. Hearts were bolus loaded over a 10min period with a combination of FluoVolt (20µL diluted in 200µL of a 5% pluronic f-127 solution) and Rhod2-AM (100µL of a 500µM stock solution) for voltage and intracellular Ca^{2+} measurements, respectively. Using this combination of fluorophores, 2P excitation at 840nm allowed voltage and calcium signals to be recorded simultaneously (detected at 510-550nm for FluoVolt and 590-650nm for Rhod2-AM). This optimized system allowed APs and Ca^{2+} transients to be recorded from deeper layers in all regions of the heart, and afforded a modest improvement in structural imaging at depth (down to ~300µm deep in infarct tissue, vs ~250µm for the LSM 510). Due to the increased average power available at 840nm (vs 920nm), power was kept low at the surface layers and adjusted automatically relative to transmural focusing depth. S/N ratios in healthy remote myocardium therefore remained relatively flat throughout the range of depths investigated (see Figure 8B).

Supplementary figure legends

Supplementary figure 1.

Signal to noise calculation of optical signals. The process for estimating signal to noise ratio (S/N) from optical action potential (AP) and Ca^{2+} transient recordings in (**A**) traces containing an AP with high signal to noise, and (**B**) myocardial layer with no detected signal. Using time averaged recordings, the amplitude of whole recording and the diastolic period were estimated for a full stimulus interval (200ms). The amplitude of both periods was divided by the standard deviation of the diastolic period, with the ratio of the resultant values representing the overall S/N.

Supplementary figure 2.

Fluorescent intensity decay with depth. A Fluorescence intensity decay profile relative to signal intensity 50µm below the epicardial surface in isolated perfused rat hearts loaded with di-4-ANEPPS (Normal zone, n=5). Photomultiplier tube gain was fixed in each heart. Mean signal intensity decay

values at each depth (black squares) could be fit with a mono-exponential decay function (red line: tau = 137±9; ymin = 43±3%). The background signal in the absence of 2P excitation is represented by the solid black line (mean) and dashed black lines (± standard error). **B** Overlay of representative traces from a single rat heart loaded with di-4-ANEPPS. Baseline values are subtracted to show signal amplitude in each trace. Signals recorded from the BZ 1000µm below the surface (green line), are nearly indistinguishable from the background fluorescence signal obtained with no excitation (black line).

Supplementary figure 1

A

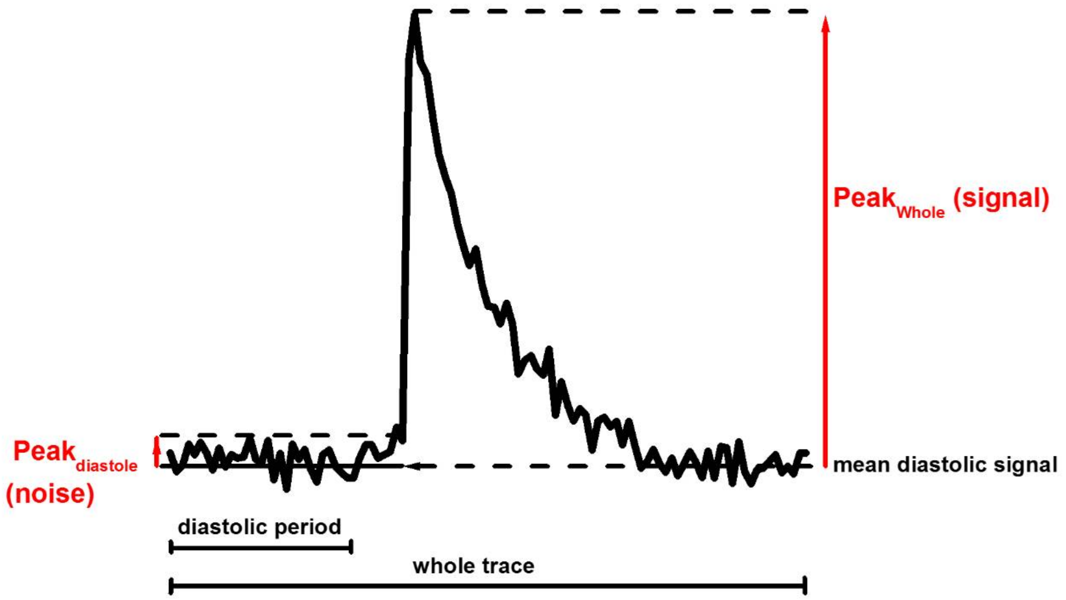

B

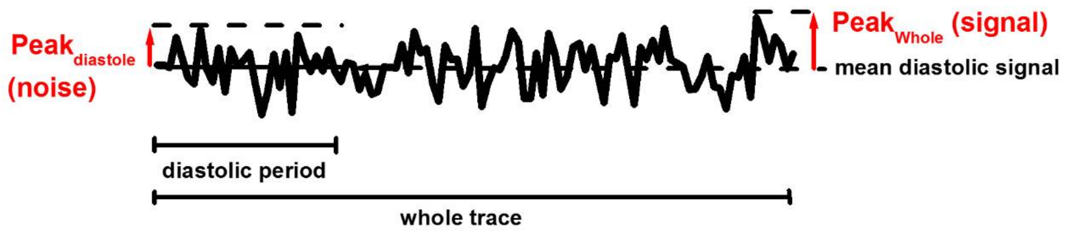

Supplementary figure 2

A

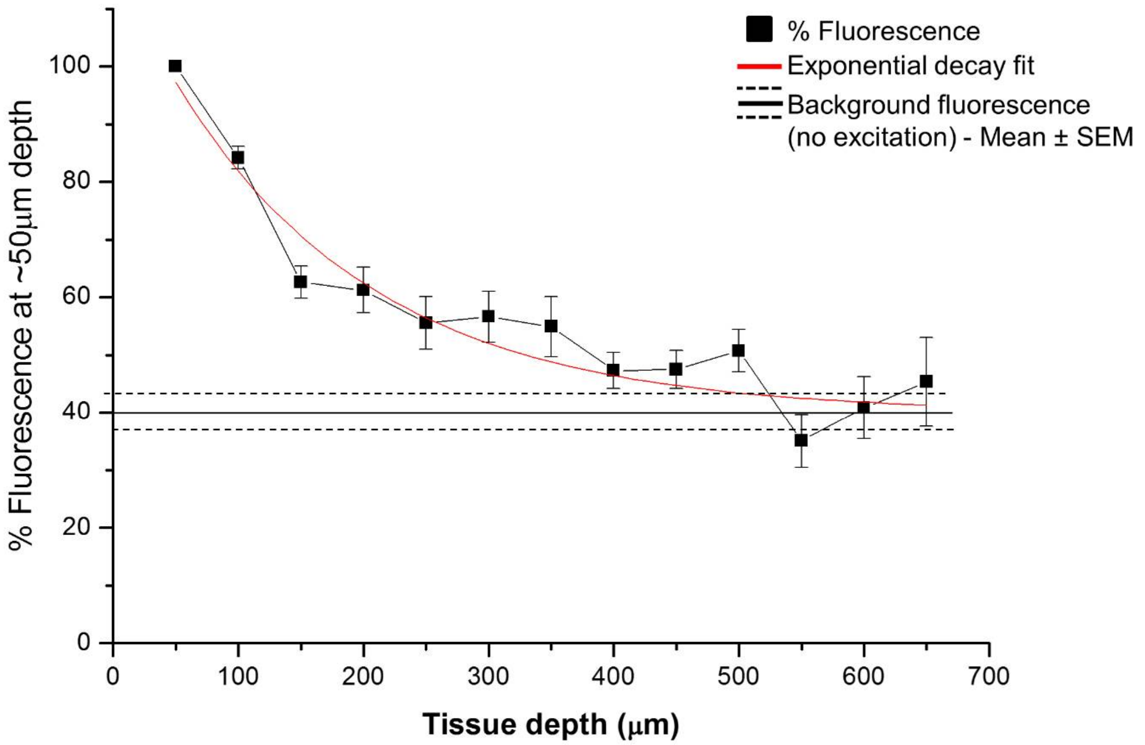

B

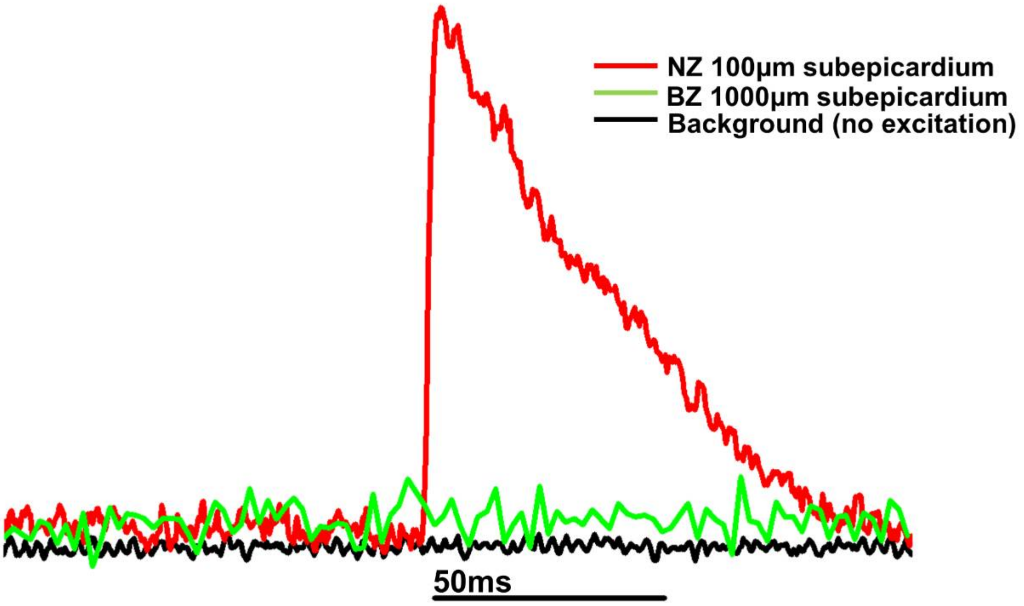

Supplement: Supplementary file 1 [file Table_1.pdf]
